# Limitations of 2-dimensional line-scan MRI for directly measuring neural activity

**DOI:** 10.1162/imag_a_00275

**Published:** 2024-08-28

**Authors:** Joshua M. Wilson, Hua Wu, Adam B. Kerr, Brian A. Wandell, Justin L. Gardner

**Affiliations:** Department of Psychology, Stanford University, Stanford, CA, United States; Wu Tsai Neurosciences Institute, Stanford University, Stanford, CA, United States; Center for Cognitive and Neurobiological Imaging, Stanford University, Stanford, CA, United States; Department of Electrical Engineering, Stanford University, Stanford, CA, United States

**Keywords:** DIANA, functional MRI, line-scan MRI, physiological noise

## Abstract

A 2D-line-scan MRI sequence has been reported to directly measure neural responses to stimuli (the “DIANA response”). Attempts to replicate the DIANA response have failed, even with higher field strength and more repetitions. Part of this discrepancy is likely due to a limited understanding of how physiological noise manifests in 2D-line-scan acquisition sequences. Specifically, it is unclear what the consequences are of breaking the assumption that the imaging substrate remains constant between each line acquisition. To answer this question, we collected 2D-line-scan data at 3T from human subjects viewing a blank screen. We found temporal fluctuations in the reconstructed time series that could easily be confused with neural responses to stimuli. These fluctuations were present both in the head and in the surrounding empty volume along the span of the phase-encoding direction from the head. The timing of these fluctuations varied systematically and smoothly along the phase-encoding direction. These artifacts are similar to well-known phase-encoding artifacts in EPI and GRE images, but are exacerbated due to longer acquisition times in the 2D-line-scan sequence (seconds vs. milliseconds). We explain these artifacts with a model that accounts for the acquisition sequence and incorporates time-varying contrast fluctuations and movement in the imaging substrate. Using the model, we quantify the amount of cortical- and scan-averaging one might need to reliably distinguish a DIANA response from noise, and show that navigator echoes might help in reducing phase-encode noise in the 2D-line-scan sequence.

## 1 Introduction

There has been a decades-long search for a noninvasive magnetic resonance method that directly measures neural responses at high spatiotemporal resolution (Bandettini et al., 2005;Bodurka & Bandettini, 2002;Petridou et al., 2006). A recent study reported that 2D-line-scan MR imaging sequences, traditionally used for high-temporal resolution cardiac and BOLD imaging (Bohning et al., 1990;Lee et al., 2021;Silva & Koretsky, 2002;Waterton et al., 1985), can record thalamic and cortical electrical responses to stimuli in mice (Toi et al., 2022). A related technique has reported responses to visual stimuli in humans (Zhang et al., 2023). The recorded MRI time series match electrophysiological measurements at millisecond resolution. However, other groups following similar protocols have been unable to replicate these results in mice and humans (Choi et al., 2024;Cloos et al., 2024;Hodono et al., 2023;Phi Van et al., 2024;Thorp, 2023).

One potential reason for this discrepancy among groups is that the signal sizes are small compared with other functional MRI measurements, such as BOLD (Ogawa et al., 1990,^1992^). Equally important, the noise characteristics of the acquisition sequence have not been well characterized, which makes it difficult to confidently discriminate signal from noise. Lack of information about the noise limits our ability to set measurement parameters such as the number of repeats and the size of spatial averaging region required to achieve high signal-to-noise ratios (SNR).

We hypothesize that the 2D-line-scan technique is particularly susceptible to phase-encoding artifacts. Unlike 1D-line-scan methods that forgo phase encoding altogether (e.g.Yu et al., 2014), the 2D-line-scan approach used inToi et al. (2022)combines phase-encoding lines, collected seconds apart, into a single k-space. The technique repeatedly acquires a single line in k-space for the duration of a single trial in the functional paradigm. Each of these lines correspond to time points only a few milliseconds apart. Every trial, a different k-space line is sampled, until all of k-space is filled for each of the time points. Depending on the trial duration and imaging matrix size, a complete measurement generally takes on the order of seconds.

Across different line acquisitions, the imaging substrate is assumed to be in the same state (stationary), thus allowing for combination of different lines of k-space into images even though they were collected at different times. It is well documented that conventional Echo-Planar Imaging (EPI) (Mansfield, 1977;Stehling et al., 1991) is subject to violations of stationarity, resulting in phase-encoding artifacts. However, because all k-space lines of a single EPI image are collected within tens of milliseconds instead of seconds, there is much less time for changes to occur than in 2D-line-scan imaging. The long 2D-line-scan image acquisition time greatly increases the chance that subject head motion, respiration, and cardiac signals (Goto et al., 2016;Greve et al., 2013;Hermes et al., 2023;Hu et al., 1995;Liu, 2016), as well as fluctuations in brain state not linked to stimulus presentation (Biswal et al., 1995;Chang et al., 2016;Lurie et al., 2020), will violate the assumption of stationarity across line acquisitions. Therefore, one would expect phase-encoding artifacts to be more severe in a 2D-line-scanning experiment.

Here we quantify the unique spatial and temporal properties of the noise in a 2D-line-scan acquisition, similar to the one used by Toi and colleagues, measured from the human brain using a 3T MRI scanner. We build a simulation that replicates the qualitative and quantitative noise profiles observed in the data. We end by quantifying how well one might be able to detect neural responses under different conditions and simulating how well navigator echoes can be used to mitigate the artifacts.

## 2 Methods

### 2.1 Participants

Five subjects (three male, two female) aged 20–35 years, naive to the goals of the study, participated in scans at the Stanford Center for Cognitive and Neurobiological Imaging. All protocols were approved beforehand by the Institutional Review Board for Human Subjects Research at Stanford University. All subjects gave informed consent prior to the start of the experiment. Data for each subject were collected in 1-hour sessions.

### 2.2 Scanner data acquisition

#### 2.2.1 Scanner and sequence

We collected data on a GE 3T UHP MR scanner with a 32-channel Nova head coil. 2D-line-scan time series data were collected using a modified SPGR-based Cine sequence (Bohning et al., 1990;Lee et al., 2021;Waterton et al., 1985); thus, the results described here do not apply to outer volume suppression (OVS) or spin-echo-based 1D-line-scan methods, in which each time point is an independent measure. A quadratic phase spoiling approach was used with a phase increment of 117 degrees.

**Fig. 1.**
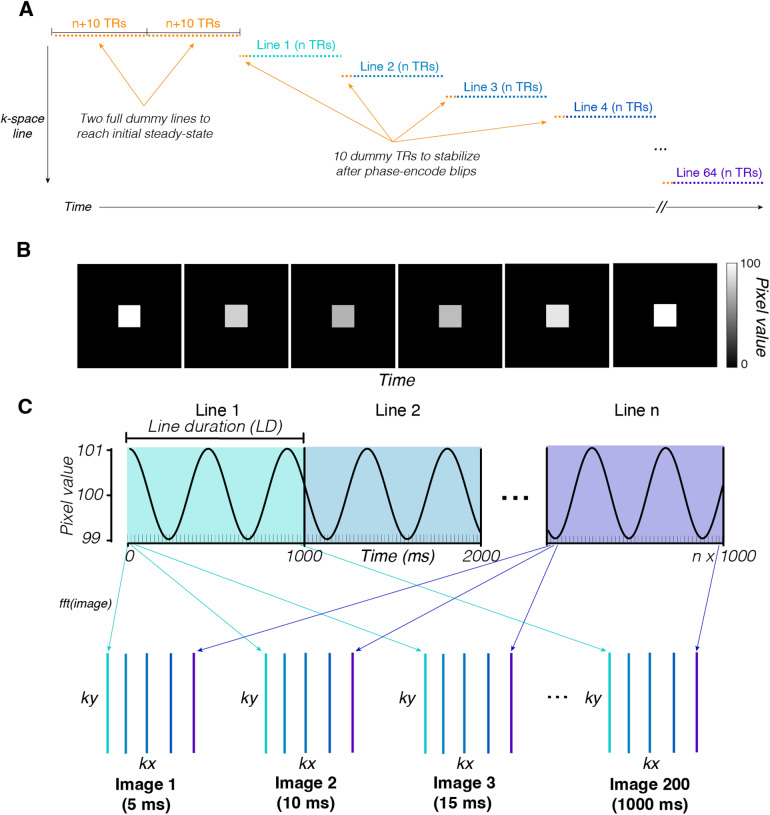
MRI acquisition and Simulation of a 2D-line-scan acquisition from a time-varying substrate. (A) MRI acquisition sequence. Each point represents a single k-space line acquisition (exact numbers labeled). (B) The input substrate is modeled as time varying; its intensity modulates as a 3 Hz sinusoid around a mean (100, 1% modulation). Voxels outside the region of the square substrate are constant at zero. (C) Line acquisitions start at random times with respect to the substrate modulation, creating nonstationarities between line acquisitions. Each line is sampled 200 times, once every 5 ms (1 second total). Data from each line acquisition are used to fill in parts of all the k-space representations in the time series. We use the inverse Fourier transform to convert the k-space acquisitions to real images.

At the beginning of the acquisition, two full dummy lines are acquired to reach steady state (360–540 TRs, depending on subject cardiac cycle length). When switching between lines, 10 dummy TRs are played out to allow for a return to steady state following phase-encoding blips (Fig. 1A). We used the same sequence parameters for both human and phantom data acquisition: a TR of 3.3 ms, TE of 1.5 ms, flip angle of 5 degrees, and bandwidth of 31.25 kHz. We collected voxels that were 3.75 × 3.75 mm in-plane and 5 mm through-plane. A single slice of 64 × 64 voxels is acquired during each scan. To avoid temporal resampling performed by the online reconstruction, all data were reconstructed offline. We did not perform any postprocessing on the data, including filtering, smoothing, or motion compensation.

#### 2.2.2 Slice selection

We selected slices with the goal of maximizing surface area of early visual cortex, where one might expect to see a strong visual response to simple stimuli (Himmelberg et al., 2022). Coronal slices were placed by hand in posterior occipital cortex in an attempt to maximize the surface area of V1–V3. A T1-weighted image was used for segmentation and surface reconstruction, which were performed using Freesurfer (Fischl, 2012). We aligned these reconstructions to a reference frame which we used to probabilistically predict early visual areas which we used in our SNR calculations (Benson & Winawer, 2018).

#### 2.2.3 Gating types and collection parameters

Eight to 10 runs were collected for each subject in each of the three different conditions: one in which the start of each line acquisition was gated to the cardiac cycle (data acquisition begins at the peak of a photoplethysmographic waveform), one in which the line acquisitions were continuous (ungated by virtue of using an emulated cardiac trigger signal), and a second ungated condition in which we swapped the frequency- and phase-encoding directions during image collection. Subject 5 did not collect data in the swapped phase-encoding condition. Because some of the scans were gated to the cardiac cycle, the number of repeated samples of each k-space line (and thus the number of reconstructed images) varied for each subject based on the length of their cardiac cycle. We collected enough repeats of each line to fill roughly 85% of the length of the cardiac cycle for each subject. Because the heart rate varies over time, it is impossible to choose a number of repetitions that both guarantees a full cycle and never misses subsequent cycles. Collecting for 85% of the average cardiac cycle length offers a good trade-off between collecting the full cardiac cycle and missing cardiac cycles. In the cardiac gated trials, if the cardiac cycle duration was more than 20% longer or shorter than the previous cycle, the line was reacquired. We collected the same amount of data in the cardiac-gated and free-running (ungated) conditions (between 594 ms and 891 ms, or 180 and 270 reconstructed images each, depending on individual subject heart rate).

#### 2.2.4 Data collection with visual stimulus

In an exploratory condition for one subject, we showed a full-contrast black-and-white checkerboard with a 1 cycle/degree spatial frequency that subtended -10 to + 10 degrees of visual angle. The checkerboard appeared 50 ms after the start of each new line acquisition, contrast reversed at 300 ms, and disappeared at 550 ms. Stimuli were generated using MGL (Gardner et al., 2018) and shown on a 60 Hz monitor. We collected 10 scans for this subject while showing these stimuli. Each scan comprised 118 images and took 63 seconds to collect the full time series (8.3 ms TR, 118 images, 64 k-space lines per image). In all other acquisitions, subjects were not shown any stimuli and were instructed to remain awake with their eyes closed for the duration of the scans. All subjects were experienced with the scanner and had partaken in previous MR experiments.

#### 2.2.5 Phantom data

We also collected data from an fBIRN agar phantom (Friedman & Glover, 2006). We used the same acquisition parameters as in the human scans and placed the coronal acquisition slice in the middle of the phantom. The data were collected continuously (ungated) and sampled 250 TRs per k-space line. We collected three scans of the phantom.

### 2.3 Temporal structure: autocorrelation and frequency analysis

To quantify the temporal structure in the data, we computed an autocorrelation permutation metric (APM). To compute the metric for each time series, we calculated the autocorrelation values of the time series: the correlation of the time series with itself at all possible time lags. We then calculated a null autocorrelation distribution for comparison which specifies the distribution of correlation values expected for any time lag of a randomly organized (temporally unstructured) time series by following the same procedure for 100 randomly permuted versions of the same time series. The autocorrelation metric for the time series is reported as the percentage of lags at which the nonpermuted cross-correlation value falls outside of the 95% bounds of the permuted distribution for the same lag. This metric is used to characterize relative levels of temporal structure between voxels rather than as a significance test.

To quantify the distribution of temporal frequency amplitudes, we averaged the amplitude spectrums of all voxels that met an autocorrelation cutoff for each subject (>70%), and then calculated the spectral components using a Fast Fourier Transform. We then averaged the first 75 frequency components across subjects, a cutoff determined by the lowest number of TRs collected in all subjects. To describe the amplitude distribution as a function of frequency, we fit curves of the form

$$Amp\left(f\right)=\frac{a}{f}+b,$$ (1)

where “a” is a scaling factor and “b” is an offset. Best-fit parameters are found via Nelder-Mead simplex algorithm. We used an adjusted r-squared to evaluate the fit of the curve that penalizes the fit for the number of parameters.

### 2.4 Simulation

Our simulation mimics how a 2D-line-scan acquisition reconstructs images. We first generate a ground-truth image time series of simple form: an image with a square “brain” (referred to as the “substrate”) in the center that modulates sinusoidally (Fig. 1B); this modulation was chosen for its simplicity and ease of parameterization, rather than a more realistic simulation of underlying physiological noise. This modulation mimics T2* contrast modulations in the human brain. In the ground-truth sequence, the substrate is a 16 × 16 square of voxels centered in a 65 × 65 image and each voxel has an average value of 100 throughout the simulation. All nonbrain voxels have values of 0 throughout the entire simulation.

We reconstructed images by building up lines of the 2-dimensional Fourier transform of subsequent images one line at a time (Fig. 1C). Our simulation reconstructs a sequence of images using parameters similar to those used in the actual data acquisition. In all simulations, we use a 1000 ms line duration, 5 ms sample rate (TR), and sample 65 lines of k-space to reconstruct a 65 × 65 × 200 sequence (x, y, t). We use 65 lines rather than the 64 used in human data acquisition, as using an odd number of lines is convenient for examining the DC component as well as ensuring that all positive and negative frequency components are complex conjugate pairs. This conjugate pairing helps implement the half-Fourier approach described below. For each sample, we take the 2-dimensional Fourier transform of the image and add one line to the corresponding k-space image. In the reconstruction, we implement a half-Fourier approach where instead of sampling all of k-space, we sample the first half and set the second half as the complex conjugate to ensure that our reconstructed images are real valued. While, in practice, 2D-line-scan images contain complex-valued components that represent phase information, the results we present do not require simulating this phase component.

We simulated substrate contrast modulation either as a simple sinusoid (with a 3 Hz frequency, phase, and amplitude of 1) or as noise with a 1/f power spectrum (pink noise). For convenience, to induce nonstationarity between line acquisitions when modulating the substrate voxels as a sinusoid, we randomized the phase of the oscillation at the beginning of each line acquisition. This offsets the phase of the modulation at each line while modulating at an integer frequency, which would otherwise not be offset as it would perfectly divide the 1-second line duration.

In addition to the 2D-line-scan acquisition simulations, we implemented a simulation in which lines of k-space were sampled in an EPI trajectory (TR = 330 ms), using the same brain modulation as in the previously described 2D-line-scan acquisition. In the EPI scan, all the lines of one image are sampled before sampling lines of the next image. The individual k-space lines were sampled equally quickly as in the 2D-line-scan acquisition (5 ms per).

In another simulation, the signal level of the simulated brain was held constant, but its position oscillated sinusoidally along the phase-encoding dimension. The simulated brain oscillation was set to a frequency of 3 Hz, the same frequency used for the contrast modulation simulation, while the amplitude was 0.5 voxels (equivalent to 1.875 mm in the actual data).

We also examined the effect of spatially localized modulations with different phases within the image. In these cases, instead of modulating all voxels in the simulated brain together, we modulated spatial groupings of voxels together with offsets between the groups. To define the spatial groups, we split the brain into equally sized rectangles along the phase-encoding direction or into squares across both directions.

Another simulation was run to show how one might correct the asynchronous contrast fluctuations using navigator echoes (Hu & Kim, 1994). When sampling each line, we also sampled the center point of k-space to measure the mean signal level of the entire imaging volume. Before combining the k-space lines in the reconstructed image, we scaled the k-space lines such that the overall image had the same mean level as the very first image in the entire series (from which we took the very first k-space line). This simulation describes a noiseless navigator echo.

We also ran simulations with noisy navigator echoes, where we added Gaussian noise onto each of the navigator estimates. The signal-to-noise-ratio (SNR) of the navigator is defined as the base substrate contrast (always 100) divided by the standard deviation of the added Gaussian noise. To quantify the amount of phase-encoding noise caused by navigator echo noise, we computed how much an imperfect navigator echo changes the substrate values in the reconstructed image (root mean squared error). This is calculated as the standard deviation of the percent contrast change in all of the individual substrate voxels across each time point in the reconstructed time series.

### 2.5 Noise and signal-to-noise estimates

It is almost always the case that experiments will average a response over contiguous voxels, for example, over a sensory area. We examined the effect of averaging across voxels on the magnitude of the noise in the human measurements. Thus, we calculated each voxel’s (all voxels from areas V1 to V3) Euclidean distance from the center of mass of that cluster of voxels and added them one-by-one by distance to an averaged time series, calculating the standard deviation of that time series at each step. We fit this relationship—between the number of voxels averaged and the standard deviation of the time series—with an exponential function of the form

$$\sigma \left(n\right)=a*\frac{1}{n^{b}},$$ (2)

where sigma is the standard deviation of the time series as a function of the number of voxels n, and “a” was fixed as the average standard deviation of the time series of all of the individual voxels in V1–V3. The parameter “b” was either fit to the data (maximizing r-squared via Nelder-Mead simplex algorithm) or fixed. In the case of completely independent voxels, we expect a square root (b = 0.5) reduction in the noise magnitude. We compared the observed noise reduction in each scan with this expectation by taking the ratio ofequation 2with the fit b value toequation 2with b = 0.5, evaluated at n equals the number of V1–V3 voxels in each subject.

We performed a similar procedure to calculate the effect of averaging over multiple scans on reducing noise. For each scan, we averaged all V1–V3 voxel time series. Then, for each subject, we averaged over an increasing number of scans similar to how we averaged over an increasing number of voxels and measured the effect on the standard deviation of the time series. Because some scans are noisier than others, for each number of scan averages n, we sampled every possible combination of n scans and took the average standard deviation of those averaged time series. Again, we fit an exponential function (eq. 2) to quantify the relationship between number of scans averaged and magnitude of the noise and compared it with a square-root-of-n noise reduction (b = 0.5), which is expected from independent scans. Here, that parameter “a” is fixed as the average standard deviation of all individual scan time series for each subject and the ratio is evaluated at n equals the number of scans collected.

To estimate the detectability of a synthetic DIANA signal, we assume an expected response to a stimulus of 0.2%, which is similar to the strongest responses described in Toi et al. To estimate the noise level as a function of how many voxels were included, we repeated the procedure described earlier in which we fit an exponential function (eq. 2) to describe the noise magnitude as a function of voxels included. Here we fit the function to all data across all subjects, instead of to individual subjects. Based on our previous analysis, we approximated the reduction in noise as a function of averaging scans as a square-root-n reduction. Together, these estimates give us a noise magnitude estimate based on the number of voxels and scans, which we can use to calculate SNR given the stimulus response estimate. We defined SNR in the impulse response case as the peak of the expected response (0.2%) divided by the standard deviation of the noise.

Because the expected response is relatively small, and because the noise in our data drops off at high frequencies, we hypothesized that it might be easier to detect neural responses that modulate at a single frequency over time (Boynton et al., 2011;Engel et al., 1994;Norcia et al., 2015;Regan, 1966). In this approach, the stimulus is modulated at a frequency outside of the dominant noise frequencies, making the neural response easier to detect. To compare this frequency approach with the impulse approach outlined above, we drew a similar SNR line as a function of scan averaging. This was done via simulation; first, we simulated 1/f noise of the same magnitude as the average scan across all subjects. We then added a sinusoidal response of the expected magnitude (0.2% signal change from peak to trough) at 5 Hz to the time series. To estimate the noise at the stimulus frequency, we took the average of the four adjacent frequency component magnitudes (-2 to +2 cycles/second) from the simulated time series. SNR was then defined as the ratio of the magnitude of the response frequency (minus the noise estimate) to this noise estimate. We averaged this value across 1000 simulations. We repeated this procedure across different number of scan averages, meaning that we reduced the 1/f noise amplitude as a square root of the theoretical number of scans included.

## 3 Results

### 3.1 Initial measurements in visual cortex: false positive responses

**Fig. 2.**
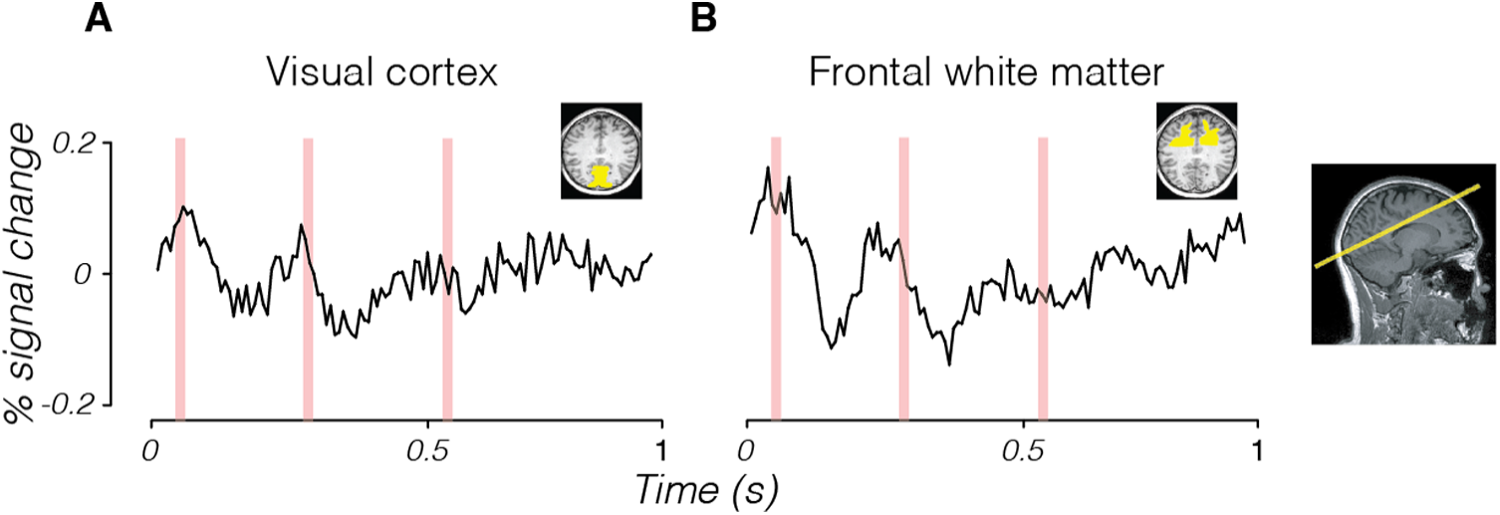
2D-line-scan time series in human visual cortex (gray matter) and frontal white matter with visual stimulus. 2D-line-scan data were collected from one subject, averaged over 10 scans. The subject viewed a flickering stimulus comprising a black-and-white checkerboard with an onset at 50 ms, a contrast reversal at 300 ms, and offset at 550 ms after the beginning of each line acquisition (times marked in red). Time series are shown as averaged across voxels in early visual cortex (A) and frontal white matter (B) (yellow shading in insets show T1-weighted slice image). The slice was acquired along the calcarine sulcus (right).

We collected 2D-line-scan data from a participant who viewed a large, high-contrast checkerboard stimulus. The stimulus was presented 50 ms after the start of each line acquisition. The stimulus contrast-reversed 250 ms after first appearing and disappeared 250 ms later. This stimulus evokes a large neural response in visual cortex (Engel et al., 1994;Gardner et al., 2008). Averaging over voxels in V1–V3, across 10 scans with identical protocol, we measured a signal comprising two peaks, roughly aligned with stimulus onset and contrast reversal (Fig. 2A).

We observed a similar pattern of activity in the time series averaged across voxels in the frontal white matter, where we do not expect to observe a neural response to the visual stimulus (Fig. 2B). This observation suggests that the fluctuations in the time series observed in early visual cortex were not stimulus-driven. We do not take this result to be a thorough replication test, likeChoi et al. (2024). Such a replication test would require, at least, demonstration of BOLD responsiveness in selected voxels, as well as an investigation into the effects of changing the stimulus and image sequence parameters. Rather, this result shows that 2D-line-scan acquisition noise fluctuations can be similar to the expected DIANA signal, and they are quite different from independent, identically distributed Gaussian noise.

### 3.2 Temporal characteristics: low-frequency modulations

#### 3.2.1 Phantom

**Fig. 3.**
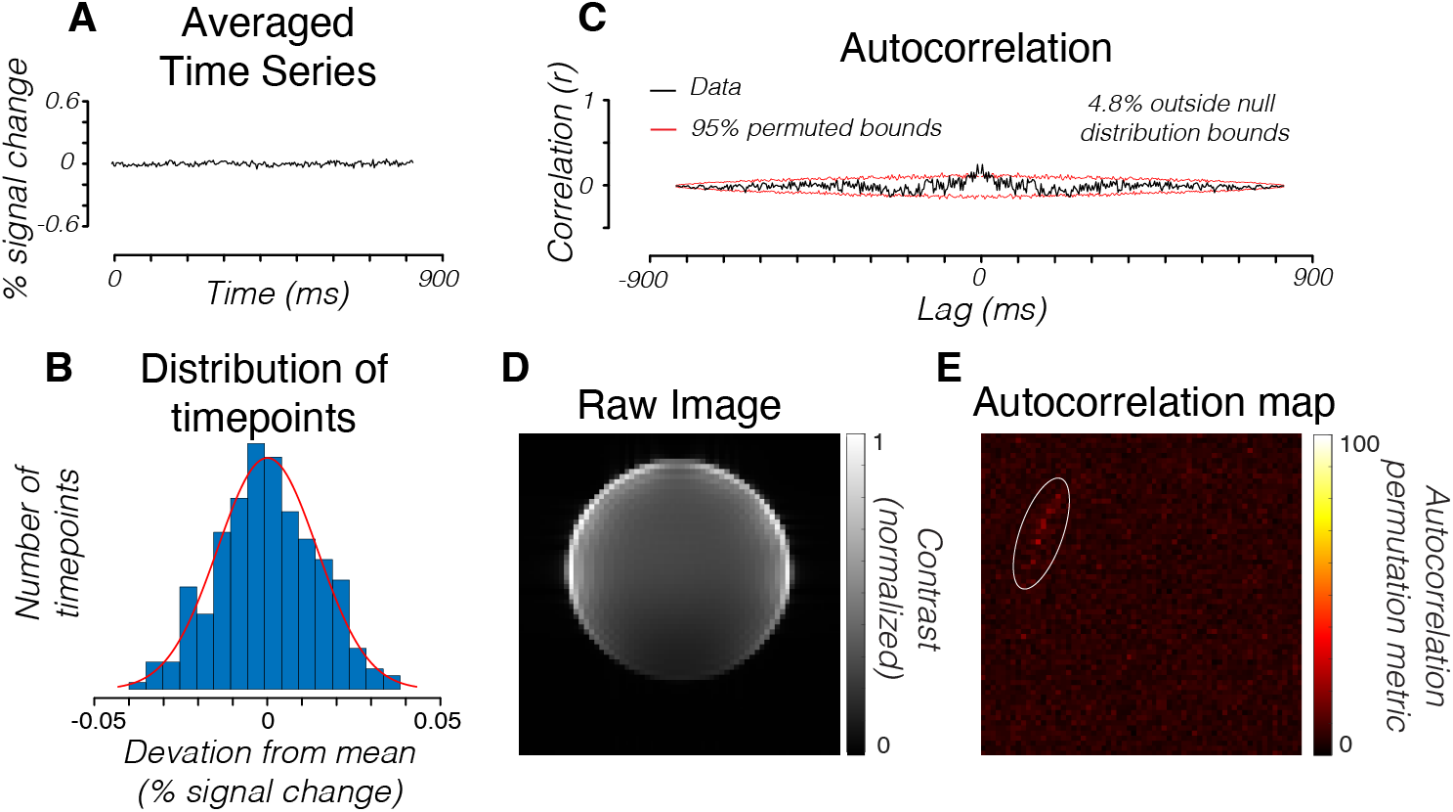
MR time series from a phantom using the 2D-line-scan protocol. (A) Average time series measured in all voxels (n = 1383) in a middle slice of an agar phantom across three repetitions. (B) Histogram of percent signal change from the mean of the data. Each observation is a single time point in the time series. Red curve is a Gaussian distribution fit. (C) The autocorrelation of the time series averaged across all voxels in the phantom. Red curves bound the confidence interval that contains 95% of the autocorrelation values computed after temporally shuffling (randomizing) the time series (100 repeats). In the phantom time series, 4.8% of the time points were outside of the 95% permuted bounds. This value is reported as the autocorrelation permutation metric (APM). (D) Mean intensity of each voxel averaged across all time points in the scan. (E) A map of the APM metric for each voxel. In the phantom, the noise is approximately Gaussian, independent, and identically distributed. The nonzero autocorrelation is very small, and appears to be due to voxels at the edge of the phantom (white circle). This may be due to small movements during the acquisition due to scanner vibrations.

To further characterize the noise and investigate the potential source of the noise, we acquired 2D-line-scan data from a slice of an agar phantom (Fig. 3A). We collected and averaged three scans using parameters similar to Toi et al. (Methods). To quantify the overall noise level, we computed the mean and standard deviation of the signal averaged across all voxels in the phantom (Fig. 3B). The time series offers a baseline measurement of the noise level. Averaged across all voxels (n = 1383), the standard deviation of all time points was 0.014% of the mean.

To quantify the temporal structure of the noise, we computed the autocorrelation permutation metric (APM) of each voxel time series and of the time series averaged across all voxels in the phantom (Methods). Were the noise independent and identically distributed, the APM would be near zero. We observed only a small deviation from this ideal in the phantom time series averaged across all voxels (Fig. 3C, 4.8% APM). Individual voxels in the phantom also had low APMs (Fig. 3E, mean = 6.03%, std = 2.01%, n = 1383 individual voxels). The location of the high-APM voxels in the phantom data—along the edge of the phantom—suggests that a central slice sets the lower noise bound in the phantom data.

#### 3.2.2 Visual cortex

To characterize the noise in humans, we acquired 2D-line-scan data from five subjects using the same parameters as the phantom. Subjects were not shown any visual stimuli and were instructed to remain awake with their eyes open for the duration of the scans. In total, 8–10 scans were collected per subject (details given insection 2.2). For each subject, we averaged together all scans and selected a region of interest in visual cortex covering V1–V3 over which we averaged voxel time series. These human-subject time series had significantly higher standard deviations than the agar phantom, even when averaged over more scans (std = 0.17%, 0.26%, 0.24%, 0.19%, 0.22%, compared with 0.014% signal change in the phantom, p < 0.001 for all subjects, one-sided*t*-test).

**Fig. 4.**
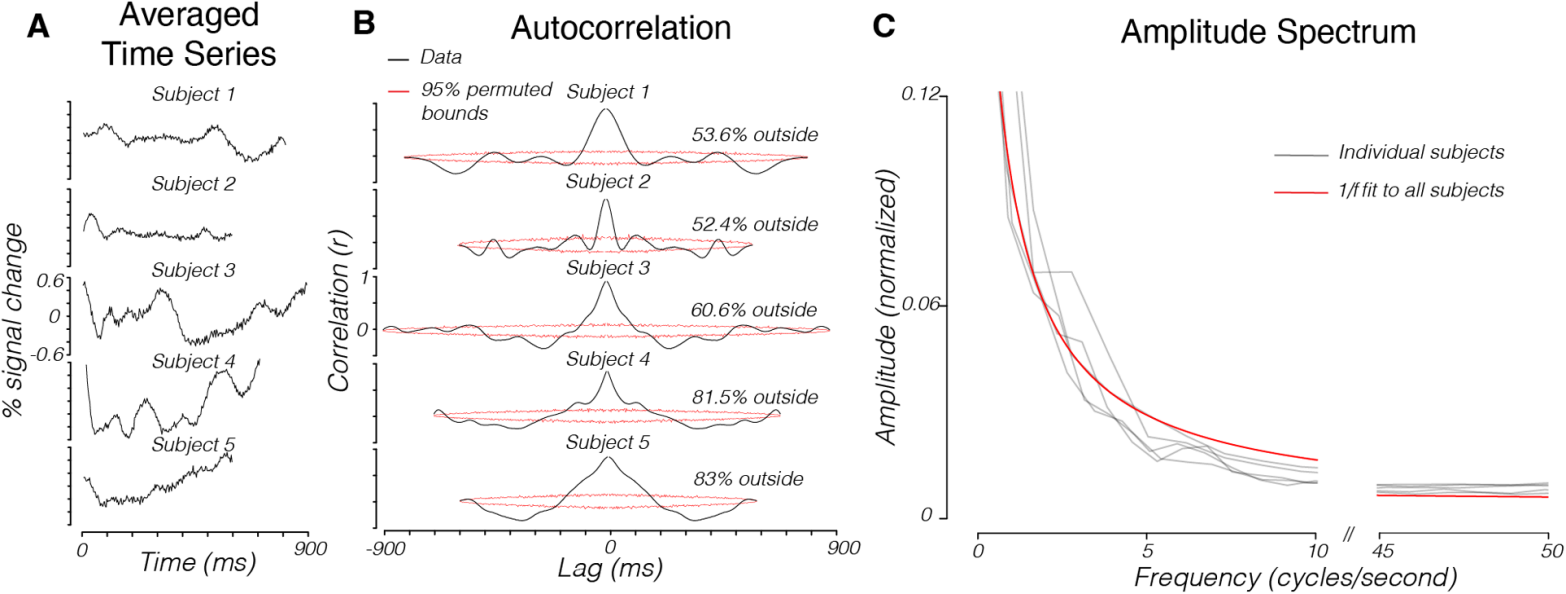
The noise distribution of 2D-line-scan time series in human visual cortex. (A) The 2D-line-scan responses for five human subjects. Each time series is averaged over V1–V3 (65–116 voxels, depending on subject) across 8–10 scans. Slices were acquired from the posterior occipital cortex. (B) Autocorrelation permutation metrics for each time series. (C) Averaged, normalized amplitude spectrums of voxels with APM values greater than 70%. Gray lines are individual subject data. Red line is a 1/f fit to all subjects’ data (eq. 1, Methods).

**Fig. 5.**
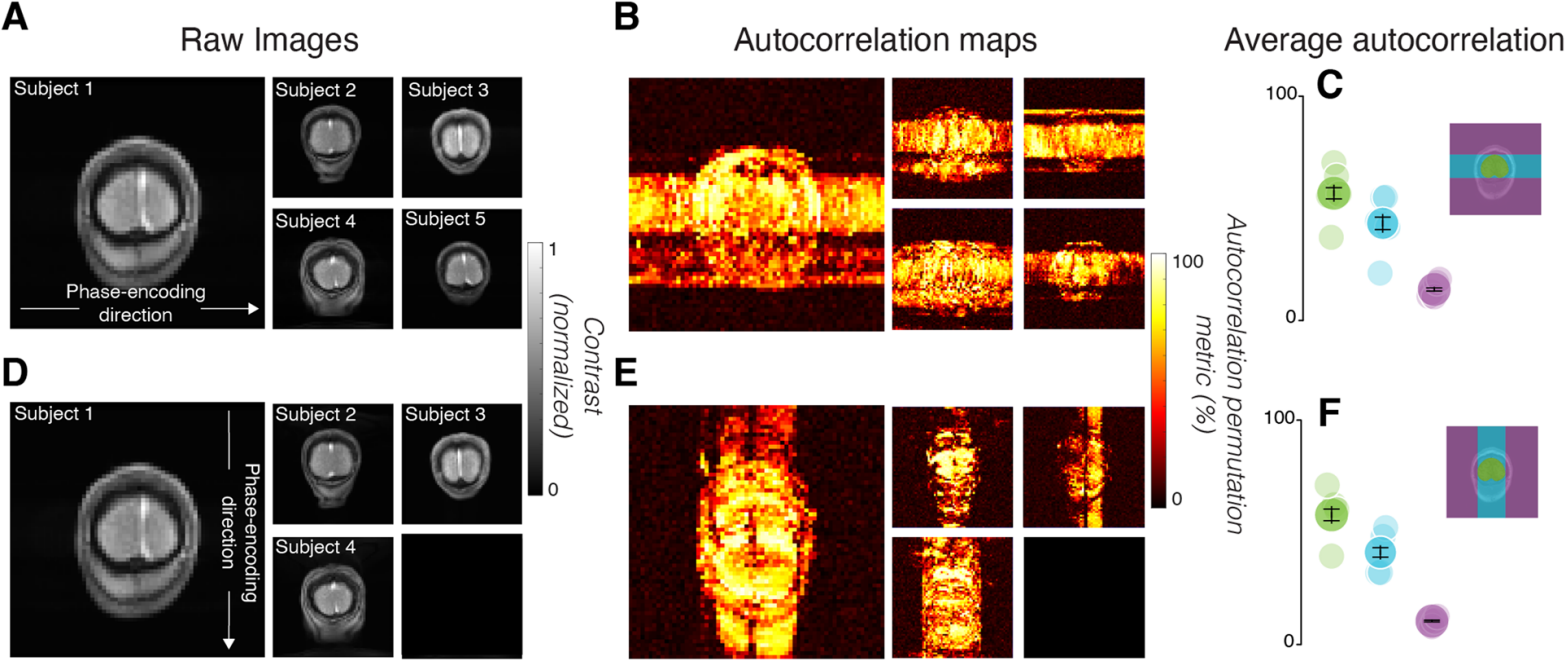
Autocorrelation maps depend on the phase-encoding direction of image acquisition. (A) Raw images acquired with the 2D-line-scan sequence for each subject, averaged over 8–10 scans and averaged across time. The phase-encoding direction was along the x-axis of the image. (B) Maps of the autocorrelation permutation metric. (C) The average autocorrelation permutation metric from In-Brain (green), Outside-Along (cyan), and Outside-Against voxels (purple), segmented for each subject (one example shown). Lighter points are individual subject data; darker points with error bars are averages of all subjects. Error bars are 1 standard error of the mean. (D–F) Same as (A–C), but with the phase-encoding direction along the y-axis. Data for subject 5 are missing in this condition due to technical error.

Unlike measurements from the phantom, the human brain time series had substantial temporal structure when averaged over areas V1–V3 (Fig. 4A), as evidenced by high autocorrelation permutation metrics (Fig. 4B) (Methods). This temporal structure was apparent when averaging the time series across voxels in early visual cortex (V1–V3) in each subject (Fig. 4B: Mean = 65.8%, std = 15.1% across five subjects). The temporal structure is also evident in individual voxels in the whole brain (Fig. 5B, E) (mean APM = 62.42%, std = 17.67%, n = 1384 total brain voxels across five subjects).

We explored the time series of all voxels with robust temporal structure (high APM values) and found that their time series have a 1/f amplitude drop-off (Fig. 4C). For each subject we normalized and averaged the amplitude spectrums of the voxels with high autocorrelation permutation metrics (> 70%) (Methods). The amplitude spectrum was well described as 1/f (Fig. 4C) (Methods,eq. 1: a = 0.22 ± 0.007, b = 0.0047 ± 0.001, adjusted r-squared = 0.94).

### 3.3 Modulations are spread along the phase-encoding direction

Voxels with high APM values were found in the brain, in the skull, and in the empty volume outside the head. Many voxels that were in line with the brain along the phase-encoding direction (along the X-axis inFig. 5B, Y-axis inFig. 5E) had high autocorrelation permutation metrics, even those outside of the head (Fig. 5B). Given this finding, we labeled voxels outside the brain along phase-encoding lines that include the brain as “Outside-Along”; voxels on PE lines that do not include the brain are referred to as “Outside-Against”; voxels inside the brain are referred to as “In-Brain” (cyan, purple, and green regions inFig. 5C). We identified and labeled these voxels by inspecting the T1-weighted image (Fig. 5A). The mean APM for In-Brain voxels was higher than the mean APM for Outside-Against voxels (Fig. 5B, C, p < 0.001, one-tailed*t*-test comparing green with purple points, mean APM = 62.15% vs. 15.28%). The mean APM for Outside-Along voxels was also higher than that for Outside-Against voxels (Fig. 5B, C, p < 0.001, one-tailed*t*-test comparing cyan with purple points, mean APM = 53.95% vs. 15.28%).

To test whether the spatial spread of temporal structure in the time series was dependent on the positioning of the phase-encoding direction, we swapped the phase- and frequency-encoding (readout) directions during image acquisition. The new Outside-Along voxels once again had a higher APM than the new Outside-Against voxels (Fig. 5E, F, p < 0.001, one-tailed*t*-test comparing cyan with purple points, mean APM = 39.76% vs. 9.37%).

**Fig. 6.**
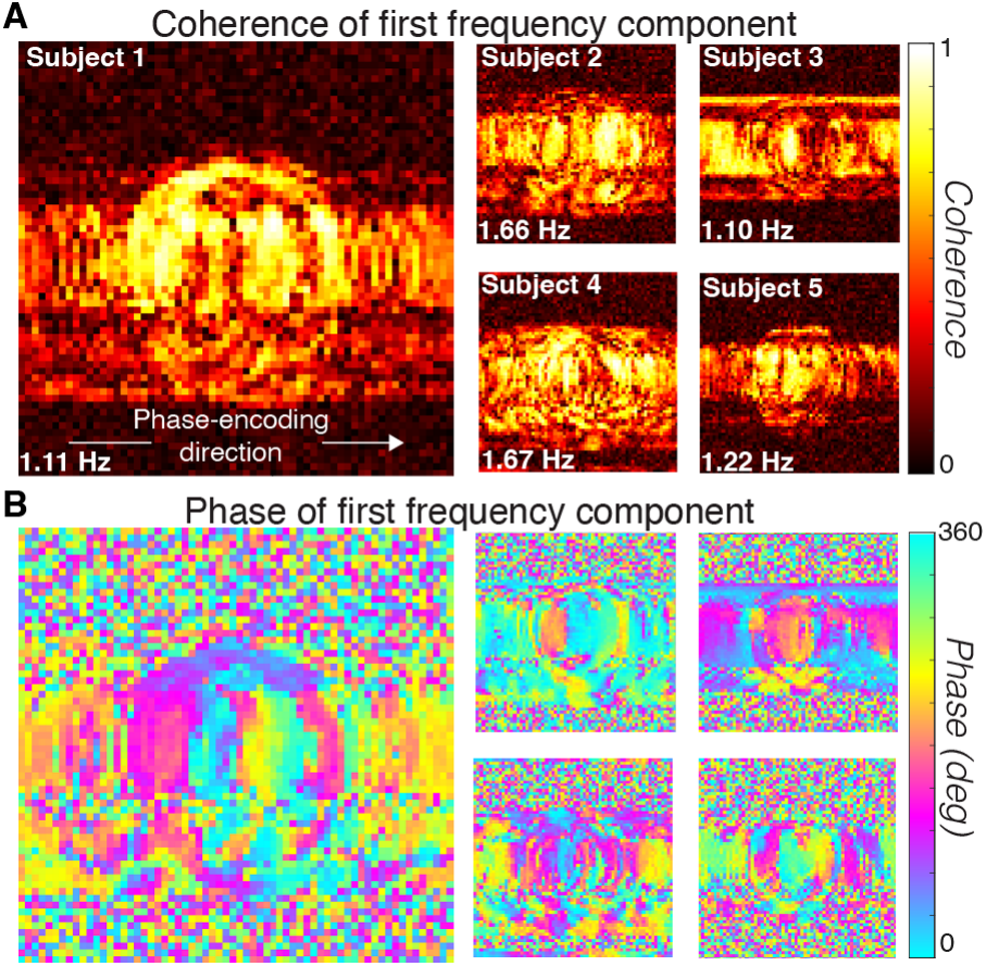
Spatial structure of the first frequency component coherence and phase. The noise is greatest in the low temporal frequency terms (Fig. 4C). Maps show the coherence (A) and phase (B) of the lowest temporal frequency (see image text). Coherence is the amplitude of the lowest temporal frequency divided by the sum of the amplitudes of all frequencies. The coherence is very low in the Outside-Against region and the phase is random across space. Within the In-Brain and Outside-Along voxels, the phase pattern for each subject is robust and approximately constant perpendicular to the phase-encoding direction.

Given the amplitude spectrum of the time series in high APM voxels, it is also possible to summarize the spatial spread using the amplitude of the lowest temporal frequency component. The image based on the coherence (amplitude at f divided by the sum of amplitudes across f) (Fig. 6A) matches the image based on the APM (Fig. 5B) (r = 0.93, 0.80, 0.91, 0.92, 0.86, pointwise Pearson’s correlation between coherence and APM maps). Thus, we can explore the spatial spread of the signal by examining both the amplitude and phase of this first frequency component (Fig. 6B).

The phase of the first frequency component reveals timing differences in the modulations across the image. The phase of the first component varies smoothly along the phase-encoding direction (Fig. 6B) across the In-Brain and Outside-Along regions. The phase is nearly constant along the direction perpendicular to the phase-encoding direction. The phase has no obvious spatial structure in the Outside-Against regions.

### 3.4 Cardiac gating did not eliminate the spatial spread

The heartbeat is one potential source of systematic temporal modulations during the acquisition, the timing of which may differ between line acquisitions. To reduce the variations on the 2D-line-scans with respect to the heartbeat, we gated the start of each line acquisition to the heartbeat while collecting data (Methods).

**Fig. 7.**
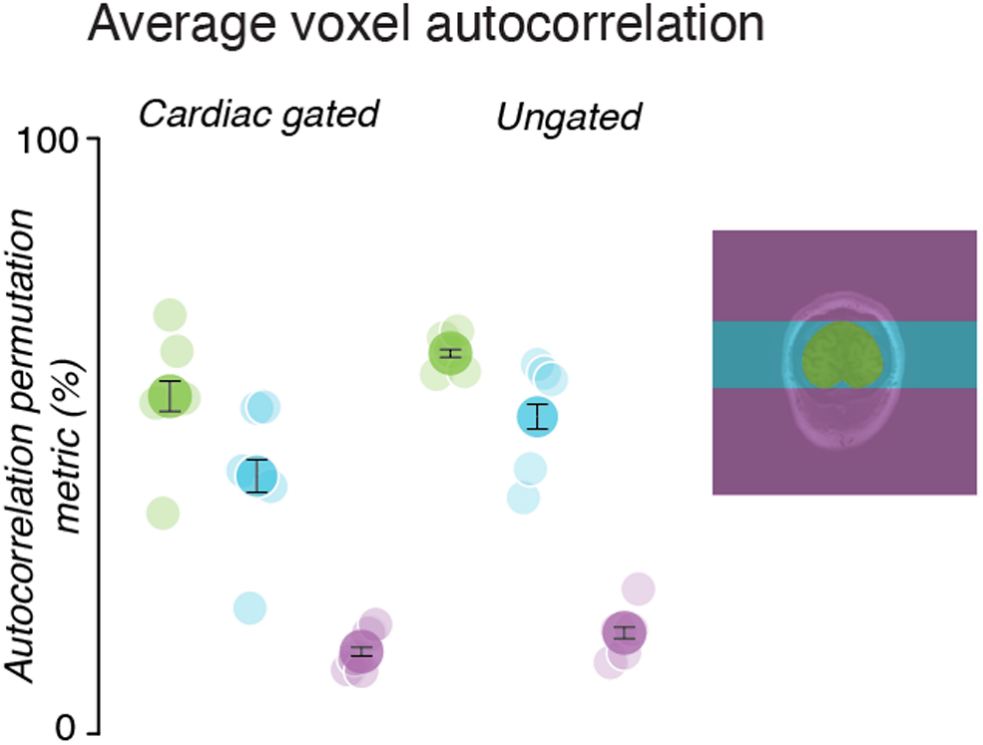
Autocorrelation permutation metrics are similar when using cardiac-gated and free-running conditions. The average APM across all subjects, along with the SEM, is shown by the darker points. Lighter points show the average APM for each subject. The colors correspond to different regions (image at right).

Gating the start of each line acquisition to the heartbeat did not significantly change the autocorrelation permutation metrics in the time series inside or outside of the brain (Fig. 7). In the cardiac-gated acquisition, In-Brain voxels (Fig. 7, left, green vs. blue, p < 0.01, paired*t*-test, n = 5) and Outside-Along voxels (Fig. 7, left, cyan vs. purple, p < 0.01) had higher APM levels than Outside-Against voxels (one-tailed*t*-tests across five subjects). However, there was no significant difference between the APM levels in the gated and ungated conditions in In-Brain voxels (Fig. 7, green points, 56% +/- 13% std vs. 62% +/- 3% std, p = 0.43, paired*t*-test, n = 5), Outside-Along voxels (cyan points, 44% +/- 15% std vs. 54% +/- 9% std, p = 0.16), or Outside-Against voxels (purple points, 14% +/- 3% std vs. 15% +/- 5% std, p = 0.38).

## 4 Model & Simulations

We built a simulation to quantify whether the artifacts can be explained by nonstationarities between line acquisitions. These artifacts are exacerbated by the long gaps between acquisitions of subsequent lines of k-space for each individual image. We simulated a 2D-line-scan acquisition of a sinusoidally modulating substrate (3 Hz, oscillating with amplitude 1 around a mean of 100), randomizing the phase of the modulation at the start of each line acquisition to induce nonstationarity between lines (Methods,Fig. 1B, C).

### 4.1 Model of 2D-line-scan acquisition recreates spatial spreading artifacts

**Fig. 8.**
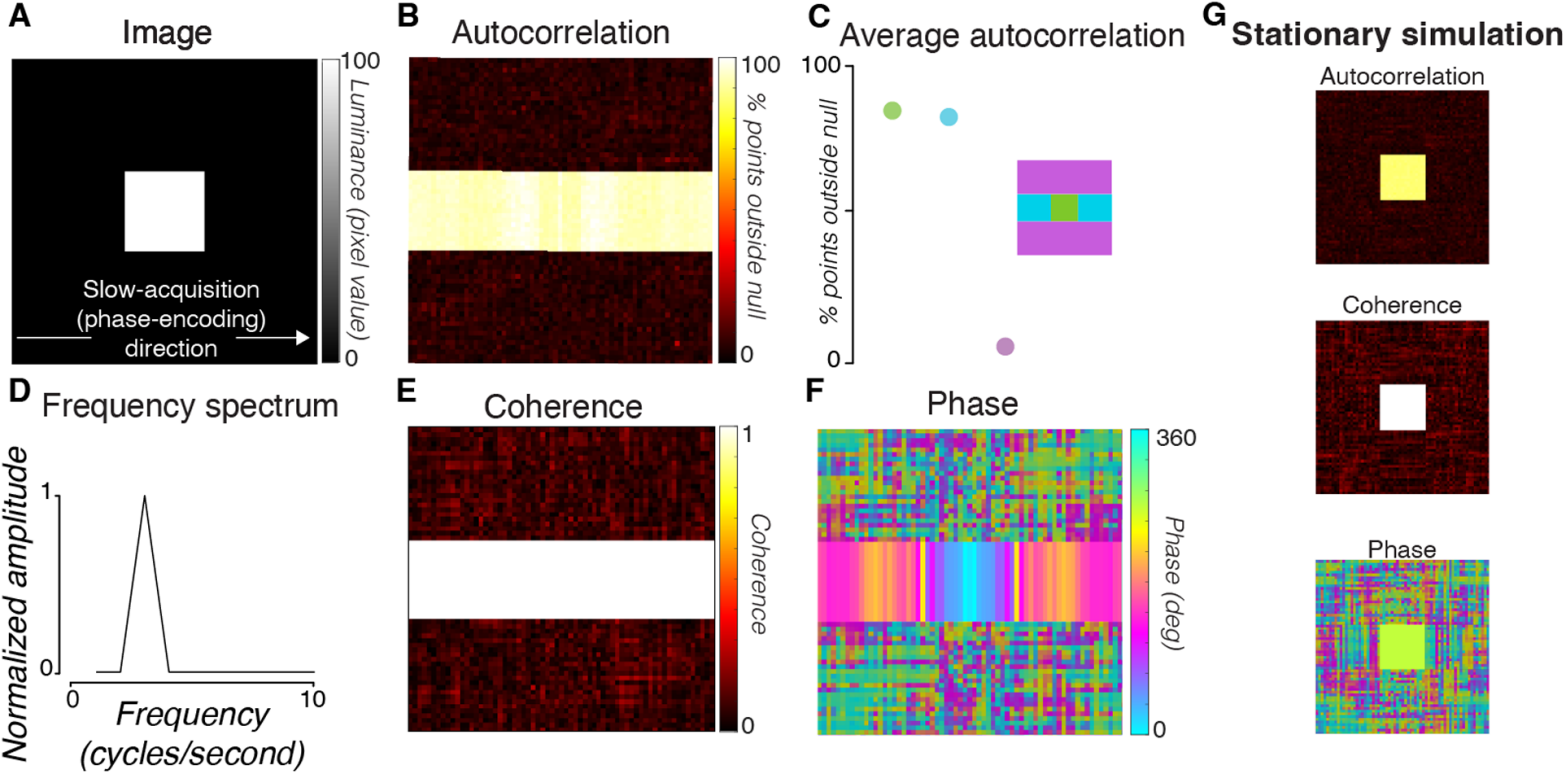
Simulation replicates spatial and temporal patterns in the data. A 2D-line-scan acquisition was simulated as the substrate intensity was modulated at 3 Hz with an amplitude of 1. The phase of the modulation was randomized at the beginning of each line acquisition. (A) Reconstructed image, averaged across all time points. (B) A map of the autocorrelation permutation metric. (C) Comparison of the autocorrelation permutation metrics in different regions of the image, shown in the inset. (D) Normalized frequency spectrum averaged over all In-Brain and Outside-Along voxels (cyan + green areas). (E) Coherence map of the substrate modulation frequency. (F) Phase map of the same component. In the simulation phase maps, we darkened the Outside-Against voxels, as their time series should be zero (any modulation is rounding error) and thus have indeterminate phases. (G) Same as (B, E, F), but for a simulation in which each 2D-line-scan was acquired at the same phase of the modulation.

The simulation predicts artifacts similar to the measurements (Fig. 8). In the simulation, brain voxels modulated over time and the voxels outside the brain were fixed to zero. Despite this, the reconstructed time series using the 2D-line-scan protocol had higher APM levels in the Outside-Along voxels than in the Outside-Against voxels (Fig. 8B/C, voxels in cyan vs. purple areas, APM = 77% vs. 7.6%, p < 0.001, one-tailed*t*-test). In the brain and Outside-Along voxels, the reconstructed response is at the modulation frequency (Fig. 8D, E). The phase of the modulation frequency varies across the phase-encoding directions (Fig. 8F), similar to the phase variation in the human data (Fig. 6B). The autocorrelation, coherence, and phase maps are symmetric across the phase-encoding direction in this simulation. This symmetry in the reconstructed images is due to the symmetry of the uniformly modulating substrate.

If each line acquisition begins at the same phase of the substrate modulation, the spatial spread is eliminated. The large autocorrelation inside the simulated head remains, and the phase is constant across the substrate (Fig. 8G). This observation motivated our experiment with cardiac gating, but the gating did not reduce the spreading in practice (Fig. 7).

When acquiring images of the same modulating substrate with an EPI trajectory, the underlying modulation interacts minimally with the acquisition sequence, causing negligible temporal modulations across the reconstructed images at the substrate modulation frequency (Supplementary Fig. 1). We observed this in a simulated EPI acquisition in which the imaging substrate was modeled the same as in the 2D-line-scan acquisition (Methods). This result highlights that slow modulations in the range produced by physiological processes like respiration or subject motion will have a much higher impact on 2D-line-scans than EPI because of the substantially longer time taken between acquisitions of each line of k-space.

### 4.2 Modulation dynamics influence the phase gradient

**Fig. 9.**
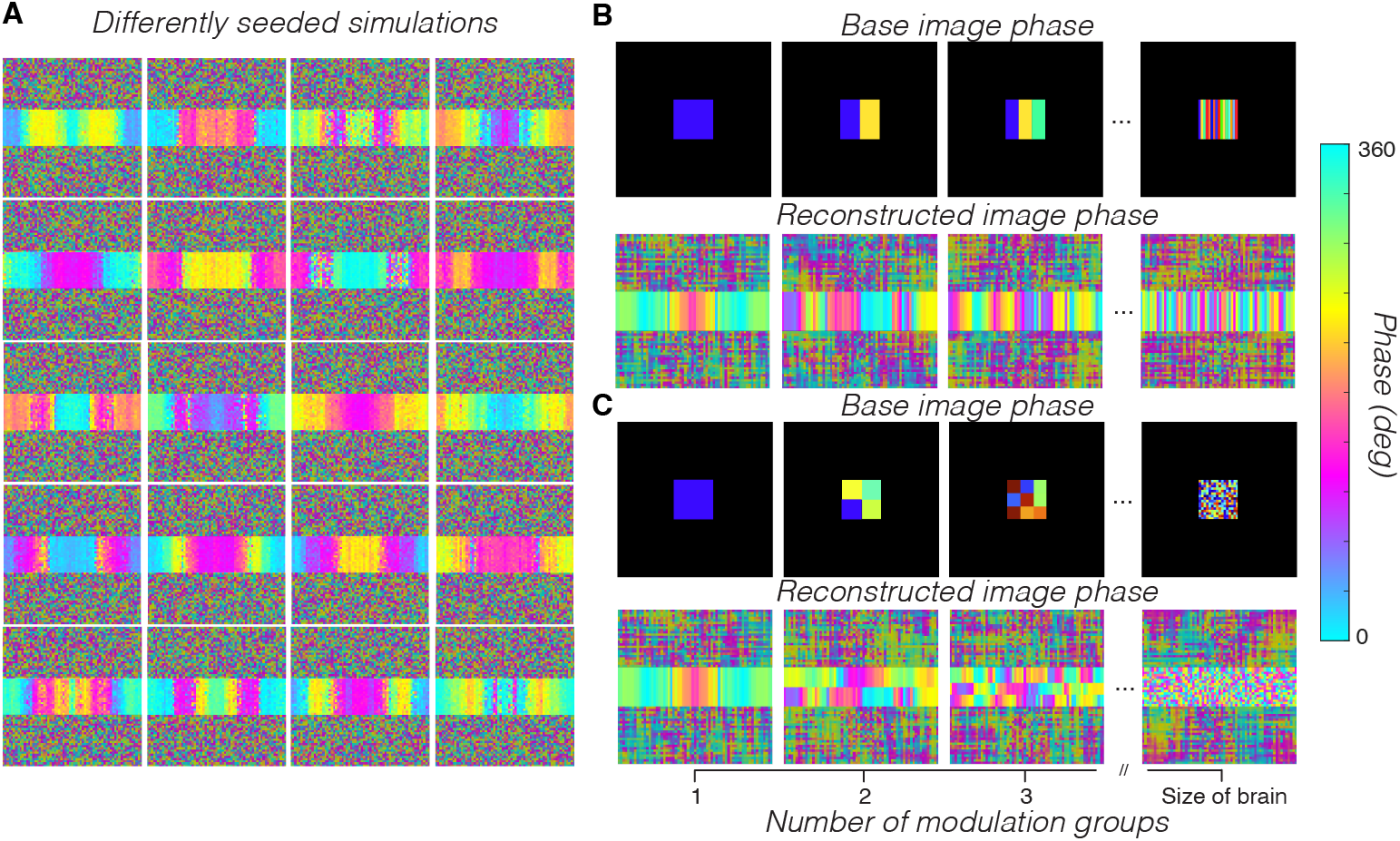
The phase map depends on the spatiotemporal distribution of the substrate modulation. (A) We simulated 20 time series, each from a 1/f noise distribution, and reconstructed the image time series using a 2D-line-scan protocol. The maps show the phase of the first frequency component. The details of the map vary between different simulations, but all the maps show the same basic pattern of spreading from the brain along the phase-encoding direction. (B, C) We then returned to using a 3 Hz substrate modulation, but varied the phase of the modulation across spatial locations. The top rows show the phase of the simulated sinusoidal modulation. The bottom rows show the phase map of the modulation frequency in the reconstructed image series. The time series of the black voxels around the simulated substrate have zero mean and zero modulation.

How the artifact appears across the reconstructed image along the phase-encoding direction depends on the offset of the imaging substrate modulation between subsequent line acquisitions. To illustrate this dependence, we ran 20 differently seeded iterations of the simulation and examined the phase gradient that emerged (Fig. 9A). Here, to simulate modulations closer to what we observed in the human brain, we modulated the ground-truth voxels globally with a 1/f drop-off in amplitude (pink noise, amplitude 1 at 1 Hz) rather than as a sinusoid. In addition, we added a small amount of independent Gaussian noise (std of 0.05% signal change) to each ground-truth voxel time series. The phase maps of the first frequency component in each simulation show how the spread of the signal is dependent on the random offset between lines, with different randomizations producing very different patterns of spread.

To determine how differences in the spatial structure of the simulated modulation change the phase of the reconstructed modulations, we ran simulations using a single sinusoidal frequency but randomizing the phase between different subregions. Breaking the assumption of global coherence in the simulation produces a progressively less-ordered phase gradient of the major frequency component along the phase-encoding dimension of the reconstructed image (Fig. 9B/C, “reconstructed image phase”). These results show that varying the global coherence of the underlying modulation in the simulation has systematic effects on the phase gradient of the artifact. Conversely, this result suggests that the observed phase gradients in the human data (Fig. 6B) may be diagnostic of the underlying modulation that is causing spreading along the phase-encoding dimension. They suggest a strong, globally coherent underlying modulation in the brain and parts of the skull.

### 4.3 Respiration as a source of artifacts: contrast modulation and motion

We have shown via simulations that asynchronous contrast modulation, perhaps due to pressure changes in the vasculature over the course of the respiratory cycle that affects T2* (Hermes et al., 2023;Shmueli et al., 2007), can give rise to the artifacts. Another possibility is that the artifacts arise from asynchronous motion related to the cardiac cycle. As the subject breathes, the brain moves both in-plane and through-plane (Terem et al., 2018).

**Fig. 10.**
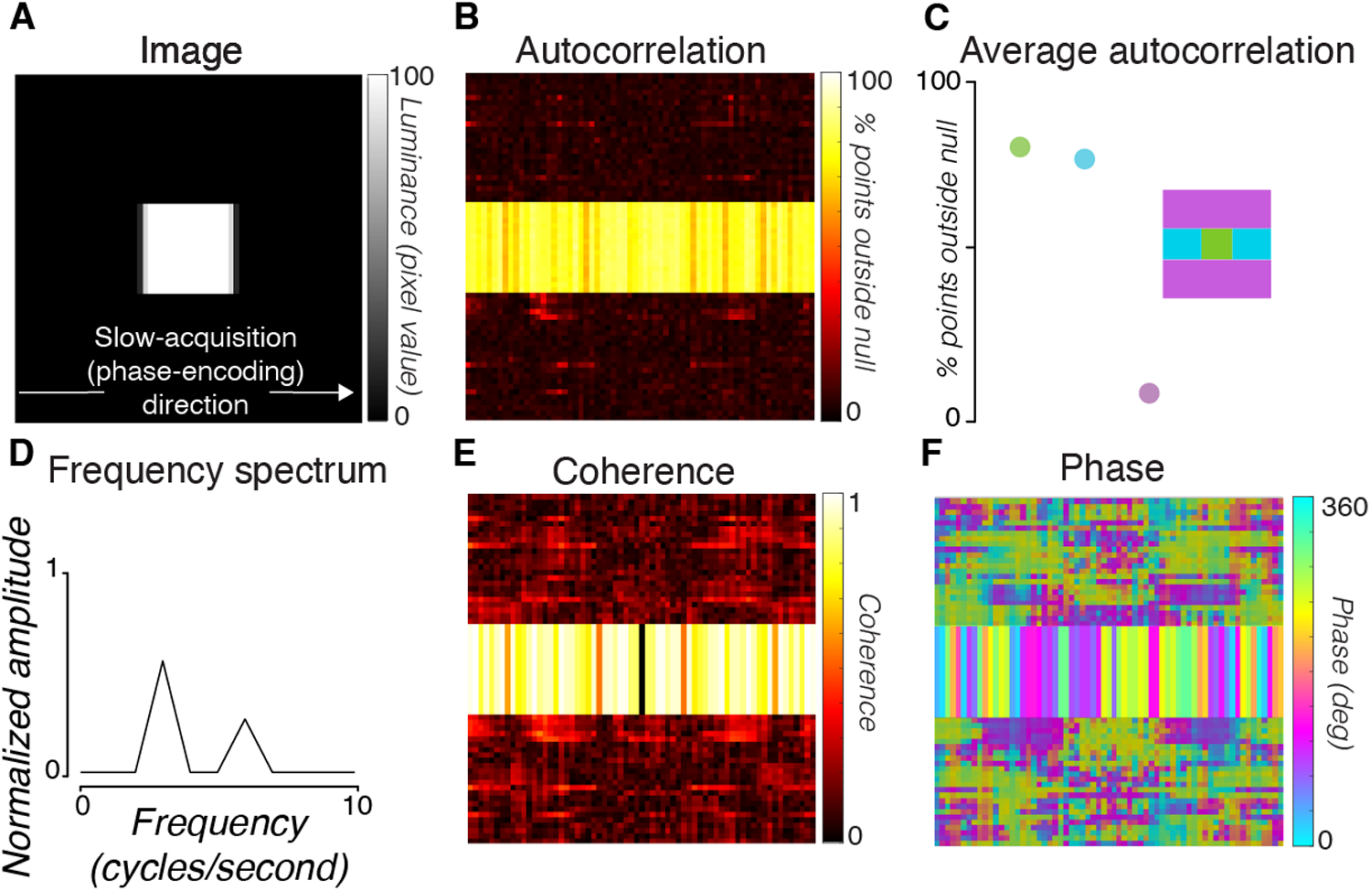
Simulation of 2D-line-scan acquisition with substrate motion. A line-scan acquisition was simulated as the substrate oscillated at 3 Hz in the phase-encoding direction. Tissue properties were constant and the simulated displacement was 0.5 voxels. The position was randomized at the beginning of each line acquisition. (A–F) Conventions are the same asFigure 8. Coherence and phase maps are shown at the movement frequency, 3 Hz.

Here we show via simulation that motion that is asynchronous between line acquisitions can give rise to the observed artifacts. We simulate a 2D-line-scan acquisition in which the substrate contrast is held constant, but the substrate is shifted sinusoidally along the phase-encoding dimension during k-space acquisition (Methods) (Fig. 10B). The phase of the motion is randomized with respect to the start of each line acquisition, the same way the phase of the contrast modulation was randomized previously. The autocorrelation spread is similar in these two cases: it spreads along the phase-encoding direction from the simulated brain. The modulation frequency of the voxels with high APM in the reconstructed images depends on the frequency of the injected motion, and also show some power at integer multiples of this frequency, because the motion was back-and-forth (Fig. 10D, E).

### 4.4 Potential for navigator echoes to correct global modulations

We simulated how a navigator echo taken before the acquisition of each individual line of k-space might reduce the artifacts observed in the data and simulation. Given that the phase-encoding artifacts in the human data appear largely global to first order, and could arise from either underlying contrast modulations (Fig. 8) or head motion (Fig. 10), it follows that they might be reduced by implementation of a navigator echo (as inHu & Kim, 1994).

**Fig. 11.**
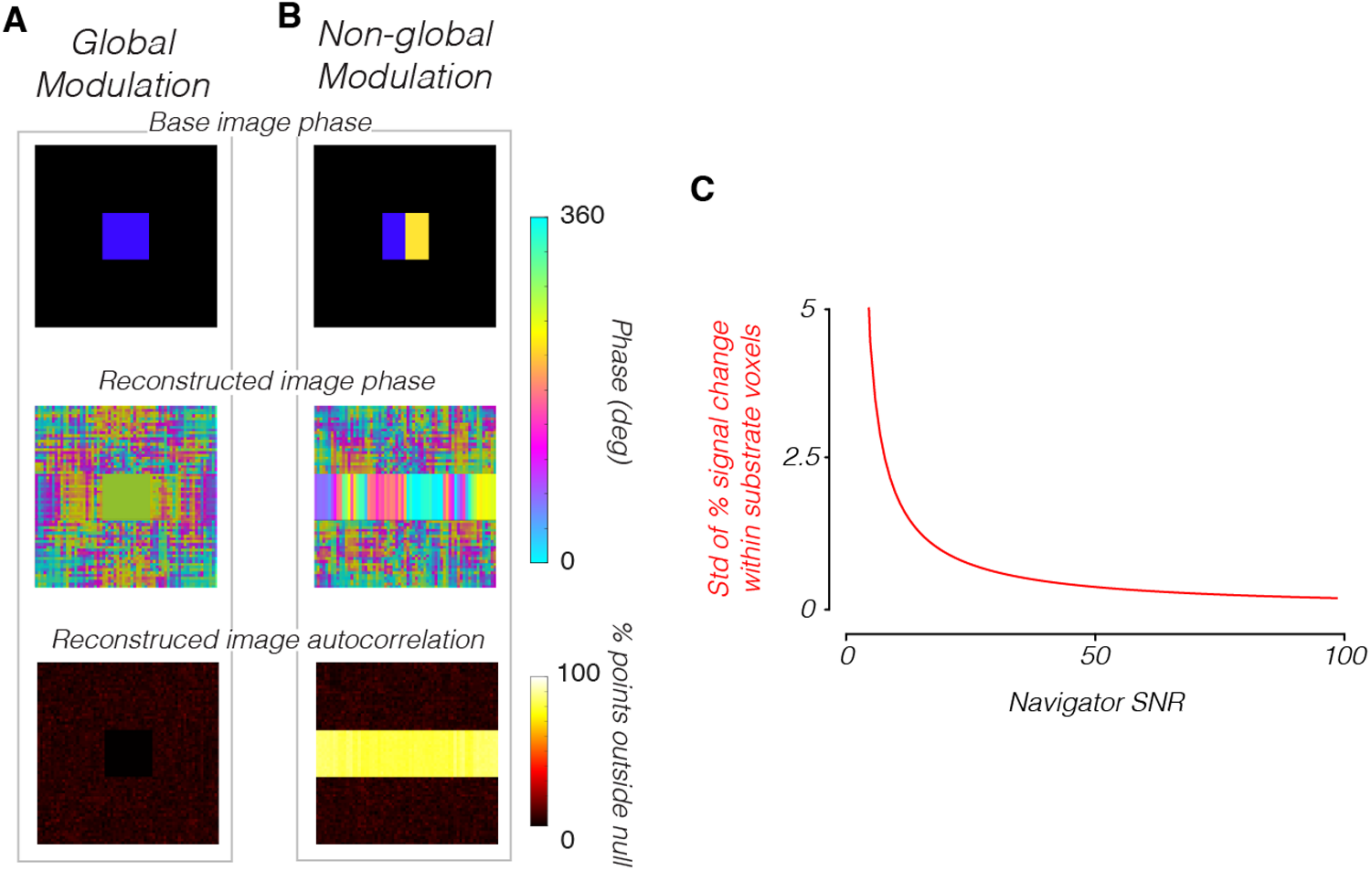
Simulation of navigator echoes. Simulations with parameters matchingFigure 9b(leftmost two columns: amplitude 1 at 3 Hz), except including navigator echoes. Voxels were modulated globally (A) or at two different phases (B). Voxels with no modulation (thus indeterminate phase) have been grayed out. (C) Quantification of phase-encoding artifacts at different navigator SNRs, with global modulation, as in (A). Standard deviation of percent signal change in voxels inside the substrate, averaged across all reconstructed images.

In the case where the modulation is global (e.g.Fig. 8), a noiseless navigator echo completely eliminates artifacts in the reconstructed image (Fig. 11A). However, when the underlying modulation is not perfectly global (modeled here as two groups of voxels modulating asynchronously,Fig. 9B), the phase-encoding artifacts remain (Fig. 11B).

In reality, navigator echoes are noisy measurements. We simulated the effect of navigator echo noise. We simulated a case of global modulation (again, same asFig. 8), implementing a navigator echo that was corrupted by various levels of Gaussian noise (Methods). As the SNR of the navigator echo decreases, the values of the substrate voxels in the reconstructed image become more noisy (Fig. 11C).

### 4.5 Expected SNR across different stimulus types and scan- and spatial-averaging choices

We used our empirical noise measurements to estimate how SNR for the 2D-line-scan technique would be expected to change for different number of trials (number of scans) and spatial (number of voxels) averaging, as well as under different modes of stimulus presentations. To do so, we calculated from our human measurements how the magnitude of the noise decreases as a function of averaging over an increasing number of scans. We calculated a similar relationship between the magnitude of the noise and the number of voxels averaged. Using these estimates of the magnitude of the noise, we calculate how many scans one must average to achieve specific SNR given the amount of cortex they are averaging over and the strength of the expected response.

#### 4.5.1 Noise reduction as a function of scan and spatial averaging

As expected, averaging over scans reduces the magnitude of the noise in the time series. We found that the average noise level of all subjects decreased from 0.55% signal change (+/- 0.06%, std across five subjects) in a single scan to 0.19% (+/- 0.05%) after averaging the scans together.

**Fig. 12.**
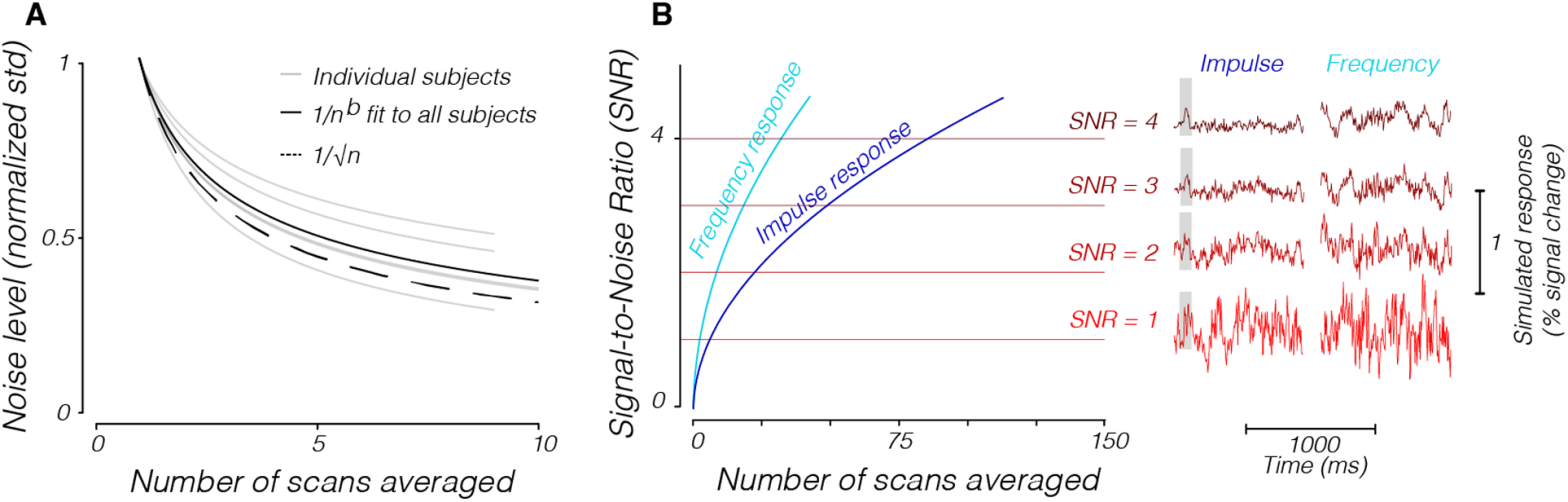
Noise magnitude and SNR estimates as a function of scan averaging. (A) The noise magnitude decreases as the number of averaged scans increases. The solid gray lines show the noise magnitude for individual subjects, and the solid black line shows the fit to all subjects. The dashed line shows the theoretical maximum noise reduction, which is the square root of the number of averaged scans. (B) The SNR increases as the number of averaged scans increases, as shown for both impulse (cyan) and frequency (dark blue, 5 Hz modulation) approaches. The right-hand side of the figure shows example time series for both approaches at certain SNRs. The time series are zero-meaned. In both cases, we assumed a single-scan noise level based on the average number of voxels in V1–V3 in our subjects (a standard deviation of 0.55% signal change from the mean).

The drop in noise magnitude for individual subjects as a function of scans averaged (gray curves,Fig. 12A) appears close to a square-root-of-n decrease (dashed black curve, adjusted r-squared = 0.82). To confirm this drop-off, for each subject, we computed a ratio of the observed noise level when averaged over all scans to the expected noise level if all scans were independent (and thus would exhibit a square-root-of-n decrease in magnitude) (Methods). When averaging across scans, the mean of the distribution of ratios across all subjects, evaluated at the maximum number of scans in each subject, was not significantly different from 1 (mean = 1.20, std = 0.24, p = 0.14, two-tailed*t*-test, n = 5 subjects), suggesting that noise between scans is independent.

Averaging over voxels in the human data also reduces the noise magnitude, but not by a square-root-of-n factor of the number of voxels included, suggesting that the variability in voxel time series is spatially correlated. Typical analyses examine contiguous voxels in particular regions of the brain and it is known that voxels are more correlated as a function of spatial proximity (Cohen & Kohn, 2011;van Bergen & Jehee, 2018). Again, we calculated the ratio of the observed noise level for all voxels averaged to an ideal noise level that decreases as square-root-of-n with voxel inclusion. The mean of the distribution of these ratios across all scans is significantly above 1 (mean = 4.68, std. = 2.46, p = < 0.001, one-tailed*t*-test, n = 46 scans), indicating that there was spatially correlated noise in the voxels.

#### 4.5.2 SNR of a response to a briefly flashed stimulus (impulse response)

Using the relationships we calculated between the amount of scan averaging and spatial averaging and the magnitude of the noise, we calculated the number of scans one would need to average over to achieve specific SNR levels when trying to measure a single-peaked response to a brief stimulus presentation (Methods). We estimated the strength of the stimulus response as 0.2% at its peak, which is close to the strongest responses reported inToi et al. (2022). To make noise estimates, we took the average noise magnitude (the standard deviation of the time series) for a single scan, averaged across all voxels in V1–V3 in our data, and scaled it based on the number of voxels and scans included (Fig. 12B).

While response magnitude and noise will vary depending on many factors, including species differences and field strengths, this plot can easily be reinterpreted to accommodate such differences. For instance, if the response magnitude is expected to be twice as large, or the noise is half of what we report here, the SNR estimates would simply need to be multiplied by a factor of 2.

#### 4.5.3 SNR of a response to a temporally modulating stimulus (frequency response)

Sinusoidally modulated stimulus designs can be constructed to improve SNR by concentrating the responses to specific frequencies outside of those in which known artifacts occur (Boynton et al., 2011;Engel et al., 1997;Norcia et al., 2015;Regan & Regan, 1988). Because the noise we measured had low amplitude in higher frequencies, a stimulus placed in those high frequencies would be more distinguishable from the noise. We calculated the number of scans one would need to average over to achieve specific SNR levels when trying to measure a response to a stimulus that is presented at a specific frequency, akin to the calculations we made for the impulse response case above (Fig. 12B) (Methods).

With the same assumptions of signal amplitude and noise estimates taken from our data, a 5 Hz sinusoidally modulated response leads to better SNR than trying to detect a single-peaked response (Fig. 12B). We note that while the BOLD response is expected to contribute very little at these higher temporal frequencies, the contribution may be nonzero (Lewis et al., 2016). Nonetheless, consideration of how the fixed hemodynamic lag would manifest in the phase of sinusoidal responses may provide a means to distinguish BOLD and other sources of signal modulation.

## 5 Discussion

While the 2D-line-scan method offers extremely high temporal resolution, it takes several orders of magnitude longer than EPI sequences to acquire any single full image. In a living animal, it is likely that there will be variations in the imaged substrate during the time required to collect a full image. Here we show that these nonstationarities cause phase-encoding artifacts that are both of greater magnitude and more structured than in EPI acquisitions. Similar phase-encoding artifacts were reported in rodents (Cloos et al., 2024). Our simulations and analysis also suggest a spatially global source, such as respiration, for these artifacts. We considered how well navigator echoes (Ehman & Felmlee, 1989;Hu & Kim, 1994) which have not been used in recent studies (Choi et al., 2024;Cloos et al., 2024;Hodono et al., 2023;Toi et al., 2022) might improve the SNR of 2D-line-scan techniques.

### 5.1 Potential physiological sources of noisy modulations in humans

The artifacts in the human data are consistent with modulations that vary smoothly across space, as evidenced from the slow changes in the phase of the first frequency component across the phase-encoding direction of the reconstructed images. The simulation supports this idea: degrading the spatial coherence of the substrate modulation (Fig. 9B) changes the spatial pattern of the phase modulations, rendering them more random. This suggests that temporal modulations in the data arise from modulations of the substrate due to biological sources such as respiration, head motion, and the flow of blood and cerebrospinal fluid.

Eliminating the spreading of these nonmovement-related modulations across the image may be impossible. Take, for example, the cardiac cycle, which induces substantial variation in the MRI signal level (Chang et al., 2009;Chen et al., 2020;Hermes et al., 2023;Shmueli et al., 2007). We found that cardiac gating did not eliminate the spreading artifacts (Fig. 7). While there may be some improvement that we were unable to detect with a small sample size, clearly the spreading effects remained even when gating to the cardiac cycle. Additionally, gating to physiological cycles that vary in length would lead to variable interstimulus intervals when attempting to measure a neural response, which may influence the neural response via adaptation.

Our data suggest that to optimize the ability of 2D-line-scan techniques to measure signals, DIANA or otherwise, the effects of modulations in the substrate must be minimized. Even if perfect gating to physiological cycles was achievable, gating would not eliminate the underlying temporal modulation in the brain. Rather, it would concentrate the modulations inside the brain instead of across the image. Because the long 2D-line-scan acquisition time makes these physiological modulations more problematic than in EPI, and because these modulations cannot be fully addressed with conventional gating, humans may be a particularly difficult species in which to measure a DIANA response.

### 5.2 Navigator echoes and noise reduction

In this study, we examined the noise characteristics of the 2D-line-scan technique in the absence of navigator echoes so that we could compare with published results of 2D-line-scan studies, which did not use navigator echoes (Choi et al., 2024;Cloos et al., 2024;Hodono et al., 2023;Toi et al., 2022). Noise-free navigator echoes (Ehman & Felmlee, 1989;Hu & Kim, 1994) eliminate phase-encoding artifacts in 2D-line-scan data in the case of global substrate modulation (Fig. 11A). However, navigator echoes are not guaranteed to improve data quality. They are ineffective at correcting for spatially localized modulations in the imaging substrate (Fig. 11B), which is the typical measurement case.

Echoes also introduce significant overhead in terms of scan timing, lowering the sampling rate significantly. Collecting an echo before each k-space line slows data collection by 10–20%. Additionally, noisy navigator echoes exacerbate phase-encoding artifacts. Careful measurement of the effectiveness of navigator echoes is required to assess their effectiveness and determine whether their benefits outweigh downsides.

### 5.3 Measuring a response

The difficulty in eliminating the consequences of substrate modulations suggests that the best approach to picking up an electrophysiological signal is to use a stimulus that evokes a response that is unlike the artifacts. This can be done by selecting appropriate stimuli. The 1/f noise structure suggests that increasing the stimulus frequency will reduce the impact of the artifact. The selection of a stimulus frequency, however, must also account for the fact that cortical neurons are better tuned to lower temporal frequencies. We find that a 5 Hz stimulus is high enough frequency to be outside of the major noise terms, and is still in the 4–10 Hz range that generates a large neural response in early visual cortex (Foster et al., 1985;Hawken et al., 1996;Horiguchi et al., 2009;Stigliani et al., 2017;Sun et al., 2007). However, sinusoidally modulated stimulus designs may also more easily confuse a potential DIANA response with BOLD signal contamination. While BOLD signal modulation is expected to decrease at higher stimulus frequency, stimulus-driven BOLD responses can still produce signal changes at high frequencies (Lewis et al., 2016).

### 5.4 Contrast adaptation and SNR

While neural responses to a repeated stimulus decline due to effects such as repetition suppression (Dobbins et al., 2004;Grill-Spector et al., 2006) or contrast adaptation (Gardner et al., 2005;Kohn & Movshon, 2003;Ohzawa et al., 1982,^1985^), our analysis and modeling suggest that the typical strategy of averaging over repeats can overcome signal loss due to adaptation. In the various attempts to measure the DIANA signal, stimuli have typically been presented very briefly (tens of milliseconds) with long intervals (hundreds of milliseconds) in between each presentation (Choi et al., 2024;Toi et al., 2022). Adaptation in sensory visual neurons can occur at multiple timescales from milliseconds (Lisberger & Movshon, 1999) to seconds (Bonds, 1991) to days (Bao & Engel, 2012;Engel, 2019) and recovery from adaptation typically occurs with time constants that are in the same order of magnitude as the rate of adaptation (for review, seeKohn (2007)). That is, given the order of magnitude difference between the length of sensory stimulation and the recovery time between presentations typically used for 2D-line-scans, responses would be expected to be nearly, if not fully, recovered by the next line and, therefore, produce negligible artifacts.

## 6 Conclusion

Direct measurement of spatially localized and temporally resolved neural activity in the living human brain would enable many new discoveries. This possibility justifies the excitement, and scrutiny, evoked by Toi et al. The failures to replicate the original findings (Cloos et al., 2024;Choi et al., 2024;Hodono et al., 2023;Phi Van et al., 2024;Thorp, 2023) prompted our analysis of the noise characteristics that one would expect using the original 2D-line-scan methods. The data suggest that our measurements were made in the presence of modulations in the brain and skull which are asynchronous between line acquisitions. The interaction between the sequence and these low-frequency-weighted temporal modulations creates image-wide artifacts that can easily be confused for neural responses.

While several of the basic features of the noise were expected, our results suggest that the long acquisition times make physiological modulations particularly problematic in 2D-line-scan MRI. The 2D-line-scan spreading artifacts are substantial but easy to diagnose. Modifications to k-space ordering and trajectories, combined with navigators and field monitoring, may help suppress the artifacts we have shown here. While we were unable to detect a neural response to a brief stimulus in humans with a 3T scanner, more controlled animal preparations with higher field strengths, more efficient stimuli, and more averaging, in conjunction with correction techniques such as navigator echoes, may yield more success.

## Data and Code Availability

Data and analyses are available athttps://osf.io/243pn/. Simulation and analysis code is available athttps://github.com/joshmw/emri. Some analysis code requires scripts fromhttps://github.com/justingardner/mrToolsandhttps://github.com/justingardner/gru.

## Author Contributions

All authors contributed to the designed experiments. J.M.W. and H.W. collected the data. J.M.W. created the simulations. J.M.W., B.A.W., and J.L.G. analyzed the data and wrote the manuscript.

## Declaration of Competing Interest

None.

## Supplementary Material

Supplementary Material
