# Supplementary figures and images for: Limitations of 2-dimensional line-scan MRI for directly measuring neural activity
Source: Imaging Neurosci (Camb). 2024 Aug 28;2:imag-2-00275. doi: 10.1162/imag_a_00275 (PMC12290851; doi:10.1162/imag_a_00275)

## Supplemental Figures

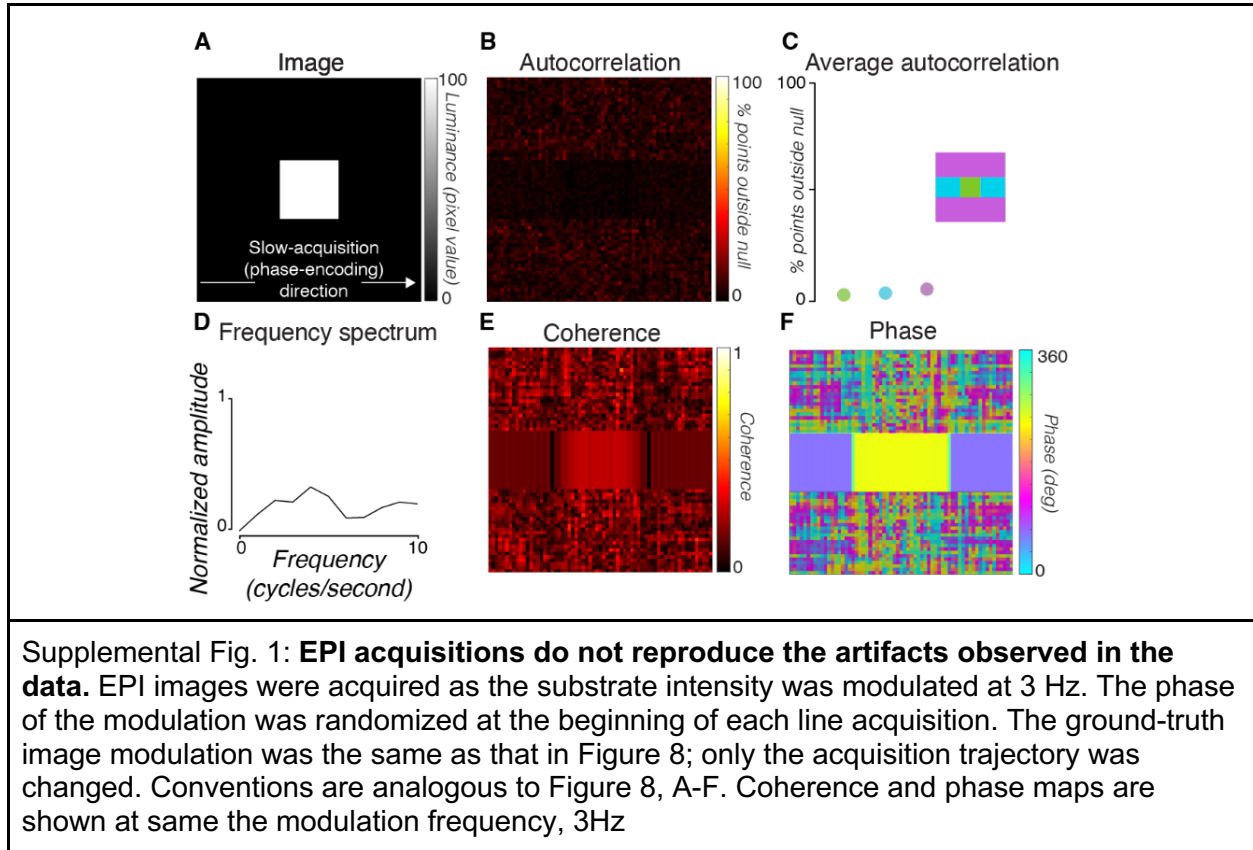

Supplement: Supplementary Material [file imag_a_00275-supp.pdf]
